# Risk of extracolonic second primary cancers following a primary colorectal cancer: a systematic review and meta-analysis

**DOI:** 10.1007/s00384-022-04105-x

**Published:** 2022-02-12

**Authors:** Dylan Robertson, Shu Kay Ng, Peter D. Baade, Alfred K. Lam

**Affiliations:** 1 School of Medicine and Dentistry, Menzies Health Institute Queensland, Griffith University, Gold Coast, QLD Australia; 2 Cancer Council Queensland, Brisbane, QLD Australia; 3 Pathology Queensland, Gold Coast University Hospital, Gold Coast, QLD Australia; 4 Pathology, School of Medicine and Dentistry, Menzies Health Institute Queensland, Griffith University, Gold Coast Campus, Southport, QLD 4222 Australia

**Keywords:** Colorectal cancer, Second primary cancer, Multiple malignancies, Risk factors, Population-based study, Meta-analysis

## Abstract

**Purpose:**

The purpose of the study is to assess the global risk of extracolonic secondary primary cancers (SPCs) in patients with colorectal cancer (CRC).

**Methods:**

Studies of SPC in patients with CRC were included if they reported the standardised incidence ratio (SIR) for extracolonic SPCs in patients with CRC compared with the general population. Pooled summary estimates were calculated using a random-effects model.

**Results:**

A total of 7,716,750 patients with CRC from 13 retrospective cohort studies that reported extracolonic SPC incidence were included. The overall risk of several SPCs was significantly higher in patients with CRC compared with the general population, including cancers of the urinary bladder (pooled SIR 1.19, 95% confidence interval (CI) 1.06–1.33; *p* = 0.003), female genital tract (1.88, 1.07–3.31; *p* = 0.03), kidney (1.50, 1.19–1.89; *p* = 0.0007), thorax (lung, bronchus and mediastinum) (1.16, 1.01–1.32; *p* = 0.03), small intestine (4.26, 2.58–7.01; *p* < 0.0001), stomach (1.22, 1.07–1.39; *p* = 0.003), and thyroid (1.40, 1.28–1.53; *p* < 0.0001), as well as melanoma (1.28, 1.01–1.62; *p* = 0.04). There was also a decreased risk of developing cancer of the gall bladder (0.75, 0.60–0.94; *p* = 0.01).

**Conclusion:**

Patients with CRC had a significantly increased risk of extracolonic SPCs compared with the general population. These findings highlight the need to develop research strategies for the management of second primary cancer in patients with CRC.

**Supplementary information:**

The online version contains supplementary material available at 10.1007/s00384-022-04105-x.

## Introduction

Colorectal cancer (CRC) is the fourth most common cancer type in the world and the third most deadly, accounting for about 10% of all incident cancers and cancer-related deaths each year [1, 2]. Although there have been improvements in the prognosis of patients with CRC due to recent advances in the screening, early detection, and treatment of CRC [3], the disease remains an important health issue worldwide. In addition, there has been an unexplained increase among young people [3–7]. This expanding population of CRC survivors faces long-term health concerns [8], such as the increased risk of developing second primary cancers (SPCs) [1, 9–21]. The reasons for this elevated risk remain unelucidated; however, various hypotheses have been posited in recent years, particularly familial genetic predispositions such as Lynch syndrome [22, 23], similar tumorigenic epigenetic changes in response to environmental exposures, or carcinogens related to tissues originating from the same germ layer [17], as well as specific mutations common to CRC and certain second cancers [24]. While the risk of synchronous and metachronous multiple malignancies of the colorectum have been well documented [20], evidence for the risk of extracolonic SPCs among CRC survivors has been less consistent [9–14, 17, 25, 26]. Around the world, CRC has been associated with extracolonic SPCs, including but not limited to malignancies of the urinary bladder [10, 12, 13, 25, 26], breast [11, 12, 27], kidney [10, 12], ovary [11, 12], pancreas [11–13, 25, 26], prostate [11, 12], stomach [11, 13, 25, 26], small intestine [11–13, 25, 26], and endometrium [12]. These mixed findings are indicative of the vast heterogeneity among countries and demonstrate the need to determine these risks to inform strategies for subsequent cancer surveillance following the management of primary CRC. Therefore, we carried out a systematic review and meta-analysis to investigate the risk of extracolonic SPCs in patients with CRC compared with the general population.

## Methods

This systematic review and meta-analysis were done according to pre-specified criteria and followed the Preferred Reporting Items for Systematic Reviews and Meta-Analyses (PRISMA) guidelines for the reporting of meta-analyses.

### Data sources and searches

We searched PubMed, Embase, Scopus, and the Cochrane electronic database for studies published from each database’s inception to 27 Dec 2021, assessing the risk of SPCs in patients with CRC, using the following search terms: “colorectal cancer”, “bowel cancer”, “second cancer”, “second primary cancer”, “second malignancies”, “multiple primary cancer”, “multiple primary malignancies”, and “multiple primaries”.

### Inclusion and exclusion criteria

Articles were eligible for inclusion if they reported the risk of extracolonic SPCs in patients with CRC, in terms of standardised incidence ratio (SIR). We included only studies that reported SIR estimates in our analyses since they provided an indirect method of adjustment for age and gender. No restrictions were applied to age, gender, comorbidities, duration, or location of the study, nor method of reporting cancer diagnoses. Articles without sufficient data, without reported individual extracolonic SPC risk, on second or multiple metachronous CRC, synchronous second or multiple cancers, centred on treatment modalities, and with overlapping populations and time periods were excluded. Only articles published in English were considered. The titles and abstracts of potentially eligible articles according to these eligibility criteria, and any duplicates, were excluded. Full-text articles were retrieved for studies that met the eligibility criteria. At this point, we excluded studies that did not include patients with CRC or did not report the SIR with respective 95% confidence intervals (CIs).

### Data extraction and quality assessment

Data were extracted from all eligible studies using predefined data extraction form: study characteristics (study design, year of publication, and corresponding author), study setting (location and period), study population characteristics (sample size, age, and gender of the patients), and outcomes (duration of follow-up and cancer incidence per cancer type). Diagnosis and confirmation of CRC and SPCs were done according to the criteria of each study. The corresponding authors of the studies, or the national registry databases used as a data source in the original studies, were consulted for additional information if required. The methodological quality evaluation of each cohort study was based on the Newcastle–Ottawa Scale.

### Outcome measures

The primary outcome measure was the incidence of extracolonic SPCs in patients with CRC, reported as SIRs. The SIR was defined in each study as the number of observed cancers in patients with CRC compared with the number of expected cancers in the general population. Specific details of how the expected number of neoplasms were calculated in each study have been summarised in Supplementary Table 1.

### Statistical analyses

We used random-effects meta-analysis to assess the risk of extracolonic SPCs in patients with CRC. To calculate the pooled SIR of SPCs, we combined the extracted study-specific estimates and corresponding 95% CIs using the DerSimonian and Laird random-effects model [28]. The Newcastle–Ottawa Scale was used to assess the risk of bias of the included studies [29]. Studies with a rating of 6 or higher were considered high quality. The heterogeneity across studies was assessed using the *I*^2^ statistic (*I*^2^ 0–25%, mild heterogeneity; *I*^2^ 25–50% moderate heterogeneity; *I*^2^ > 50%, large heterogeneity) [19]. We used funnel plots to assess the potential for small-study effects (publication bias). All statistical analyses used RevMan (version 5.4.1. Copenhagen: The Nordic Cochrane Centre, The Cochrane Collaboration). All statistical tests used a two-sided *α* value of 0.05 for statistical significance.

## Results

### Literature search

**Fig. 1:**
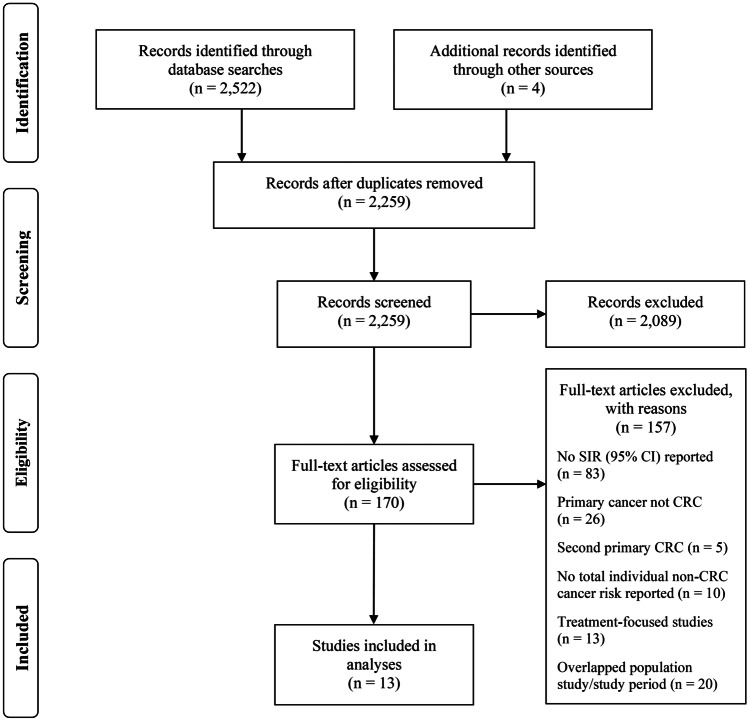
Preferred Reporting Items for Systematic Reviews and Meta-Analyses (PRISMA) flowchart

Searches returned 2522 records, with an additional 4 records identified through reference lists, of which 2259 were excluded after an initial screening of duplicates, titles, and abstracts. Full texts were retrieved for 170 studies and assessed for eligibility (Fig. 1). Thirteen studies published between 1999 and 2021, including 7,716,750 patients (2.01 × 10^9^ person-years) with CRC that reported extracolonic SPCs cancer incidence, were included in the meta-analysis according to our inclusion criteria [16, 18, 21, 27, 30–38]. The median Newcastle–Ottawa rating for the studies included was 8 (interquartile range (IQR) (7–8)). The population characteristics and outcomes of the included studies are summarised in Table 1. The median age of the study populations ranges from 56 to 73.

**Table 1:** Characteristics and quality of included studies

**Study ID**	**Study type**	**Country and data source**	**Study population, definition, and inclusion criteria**	**Primary cancer diagnosis timeframe and follow-up duration**	**Sample size** *(N)*	**Men**	**Women**	**Age (years)**	**Second primary cancer type(s); number of cases**	**Second primary cancers SIR (95% CI)**	Newcastle–Ottawa Scale rating*
Bright et al. [16]	Retrospective cohort study	England and Wales; Office for National Statistics and Welsh Cancer registry	Men and women with > 5-year diagnosis of cancer; aged 15–39 years	1971–2006; 16.8 years (median)	200,945	76,666 (38.0%)	124,279 (62.0%)	15–39	Breast: 74 Lung and bronchus: 48 Urinary bladder: 32 Prostate: 33 Melanoma: 20 Ovary: 19 Oral: 16 Corpus uteri: 68 Kidney: 23 Non-Hodgkin lymphoma: 19 Brain: 21 Oesophagus: 12 Pancreas: 9 Other female genital: 27 Stomach: 14 Leukaemia: 9	1.80 (1.06–3.07) 1.30 (0.90–1.70) 2.10 (1.40–2.90) 1.20 (0.80–1.70) 1.50 (0.90–1.10) 1.90 (1.10–2.9) 1.80 (1.00–2.90) 7.20 (5.60–9.10) 3.00 (1.90–4.50) 1.50 (0.90–2.30) 3.00 (1.80–4.60) 1.60 (0.80–2.90) 1.30 (0.06–2.50) 3.30 (2.20–4.80) 2.00 (1.10–3.40) 1.30 (0.60–1.30)	7
Caini et al. [30]	Retrospective cohort study	Italy; European Institute of Oncology database	Men and women with diagnosis of non-cutaneous malignancy	2000–2010; 4 years (median)	52,354	15,706 (30.0%)	36,648 (70.0%)	56 (median)	Melanoma: 9	1.37 (0.71–2.63)	4
Chung et al. [31]	Retrospective cohort study	South Korea; Cancer Registry database at Severance Hospital, Seoul, Korea	Men and women with a diagnosis of CRC; aged 45–74 years	2001–2009; 40.1 months (median)	4822	2981 (61.8)	1841 (38.2)	61 (median)	Pancreas: 13	14.44 (12.71–16.16)	5
Cluze et al. [32]	Retrospective cohort study	France; Cancer Registry of Isère	Men and women with a diagnosis of breast, prostate, or colorectal cancer; aged > 15 years	1989–1997; 3.5 years (mean)	14,353	6314 (44.0%)	8039 (56.0%)	66.4 (mean)	Upper aerodigestive tract: 10 Oesophagus: 3 Stomach: 3 Small intestine: 5 Liver and hepatic ducts: 3 Pancreas: 0 Lung: 16 Skin: 9 Breast: 18 Female genitals: 5 Prostate: 25 Kidney: 5 Urinary bladder: 9	1.33 (0.69–2.33) 1.10 (0.23–3.22) 0.70 (0.23–1.64) 10.70 (3.47–24.97) 0.70 (0.23–1.63) 0.23 (0.01–1.29) 1.21 (0.77–1.79) 1.52 (0.92–2.38) 1.22 (0.77–1.85) 1.00 (0.40–2.06) 1.11 (0.79–1.53) 1.90 (0.76–3.92) 1.40 (0.72–2.44)	8
Crocetti et al. [36]	Retrospective cohort study	Italy; Italian cancer registries	Men and women with a diagnosis of thyroid cancer; aged < 85 years	1998–2012; < 7 years (median)	6,984,420	3,340,798 (47.8%)	3,643,622 (52.2%)	< 85	Thyroid: 230	1.40 (1.30–1.60)	8
Dasgupta et al. [18]	Retrospective cohort study	Australia; Queensland Cancer Registry	Men and women with a diagnosis of invasive CRC; aged 20–79 years	1996–2005; 4.2 years (median)	15,755	9091 (57.7%)	6664 (42.3%)	64 (mean)	Stomach: 38 Small intestine: 20 Pancreas: 33 Lung: 202 Melanoma: 168 Breast (female): 115 Uterus: 25 Prostate: 265 Kidney: 57 Urinary bladder: 73 Non-Hodgkin lymphoma: 42 Myeloma: 21	1.43 (1.01–1.97) 4.84 (2.96–7.48) 1.19 (0.82–1.67) 1.40 (1.22–1.61) 1.37 (1.17–1.59) 1.22 (1.01–1.47) 1.57 (1.01–2.31) 1.14 (1.01–1.29) 1.61 (1.21–2.07) 1.25 (0.98–1.57) 1.02 (0.73–1.38) 1.32 (0.82–2.02)	8
He et al. [33]	Retrospective cohort study	USA; National Cancer Institute SEER database	Men and women with a diagnosis of CRC; Aged > 18 years	1973–2013; 7.3 years (mean)	44,106	25,514 (58.0%)	18,592 (42.0%)	> 18	Oropharynx: 999 Oesophagus: 576 Stomach: 1225 Small intestine: 585 Liver: 371 Gallbladder: 106 Pancreas: 1478 Lung and bronchus: 7400 Melanoma: 1254 Breast: 4949 Cervix uteri: 166 Corpus uteri: 1273 Ovary: 525 Prostate: 8101 Urinary bladder: 2947 Kidney: 68 Brain: 360 Thyroid: 410 Myeloma: 611 Leukaemia: 1306	0.95 (0.90–1.02) 1.08 (1.00–1.17) 1.16 (1.09–1.22) 3.13 (2.89–3.40) 0.76 (0.69–0.85) 0.65 (0.54–0.79) 0.99 (0.94–1.04) 1.00 (0.98–1.03) 0.89 (0.85–0.95) 0.99 (0.96–1.02) 0.96 (0.82–1.12) 1.22 (1.15–1.29) 0.88 (0.81–0.96) 0.91 (0.89–0.93) 1.00 (0.97–1.04) 1.07 (1.01–1.13) 0.85 (0.77–0.95) 1.30 (1.18–1.43) 0.86 (0.79–0.93) 0.90 (0.85–0.95)	9
Lee et al. [34]	Retrospective cohort study	Taiwan; Taiwan’s National Health Insurance Database	Men and women with a diagnosis of CRC	1996–2011; 4.03 years (median)	98,876	55,729 (56.4%)	43,147 (43.6%)	67 (median)	Oesophagus: 77 Stomach: 299 Liver/biliary tract: 636 Pancreas: 100 Lung and mediastinum: 843 Skin: 127 Breast: 275 Women genital: 257 Cervix uteri: 92 Corpus uteri: 106 Ovary: 59 Prostate: 455 Urinary bladder: 259 Kidney: 191 Thyroid: 73	0.87 (0.68–1.08) 1.02 (0.91–1.14) 0.90 (0.83–0.97) 1.01 (0.82–1.23) 1.18 (1.10–1.26) 1.12 (0.93–1.33) 1.20 (1.06–1.25) 1.64 (1.45–1.85) 0.98 (0.79–1.20) 0.32 (2.62–3.87) 2.00 (1.52–2.57) 1.19 (1.09–1.31) 1.31 (1.16–1.48) 1.45 (1.25–1.67) 1.71 (1.34–2.15)	8
Levi et al. [21]	Retrospective cohort study	Switzerland; Vaud Cancer Registry	Men and women with a diagnosis of CRC or adenomatous polyps	1974–1994; average follow-up unknown	5261	.	.	.	Oropharynx: 16 Oesophagus: 18 Stomach: 32 Small intestine: 4 Liver: 6 Gallbladder: 6 Pancreas: 6 Lung: 50 Melanoma: 12 Breast (female): 80 Cervix uteri: 10 Corpus uteri: 8 Ovary: 2 Prostate: 96 Urinary bladder: 22 Kidney: 16 Non-Hodgkin lymphoma: 12 Leukaemia: 8	0.84 (0.40–1.60) 1.36 (0.60–2.60) 1.16 (0.70–1.90) 1.89 (0.20–6.80) 0.63 (0.10–1.80) 0.71 (0.10–2.10) 0.29 (0.10–0.90) 0.70 (0.50–1.00) 0.96 (0.40–2.10) 1.29 (0.90–1.80) 1.73 (0.60–4.00) 0.63 (0.20–1.60) 0.21 (0.00–1.20) 1.19 (0.90–1.60) 0.88 (0.50–1.60) 1.25 (0.50–2.50) 0.84 (0.30–1.80) 0.59 (0.20–1.50)	8
Utada et al. [27]	Retrospective cohort study	Japan; Nagasaki Prefecture Cancer Registry	Men and women with a diagnosis of primary cancer	1985–2007; 4.3 years (mean)	174,477	.	.	.	Stomach: 751 Pancreas: 137 Ovary: 34	1.37 (1.28–1.47) 1.21 (1.02–1.43) 1.83 (1.27–2.56)	7
Ye et al. [35]	Retrospective cohort study	Australia; Tasmanian Cancer Registry	Men and women with a diagnosis of cancer > 2 months; aged > 15 years	1980–2009; 6.9 years (mean)	51,802	28,242 (54.5%)	23,560 (45.5%)	66.2 (median)	Lung: 121 Skin: 80 Prostate: 182	1.13 (0.80–1.58) 1.88 (1.38–2.55) 1.15 (0.90–1.48)	8
Zheng et al. [37]	Retrospective cohort study	Sweden; Swedish Cancer Registry	Men and women with a diagnosis of bladder or upper urinary tract cancer	1990–2015; average follow-up unknown	49,584	36,614 (74%)	12,970 (26.0%)	73 (median)	Urinary bladder: 521 Kidney: 22	1.11 (1.02 –1.21) 1.32 (0.83–1.54)	8
Zheng et al. [38]	Retrospective cohort study	Sweden; Swedish Cancer Registry	Men and women with a diagnosis of hepatobiliary cancer	1990–2015; 36 months (median)	19,995	10,102 (64.9%)	9893 (49.5%)	72 (median)	Gallbladder: 44 Bile duct: 61	0.85 (0.61–1.14) 1.20 (0.92–1.54)	8

*Newcastle–Ottawa Scale ratings ≥ 6 were considered high quality

### Risk of extracolonic SPCs in CRC patients

**Fig. 2:**
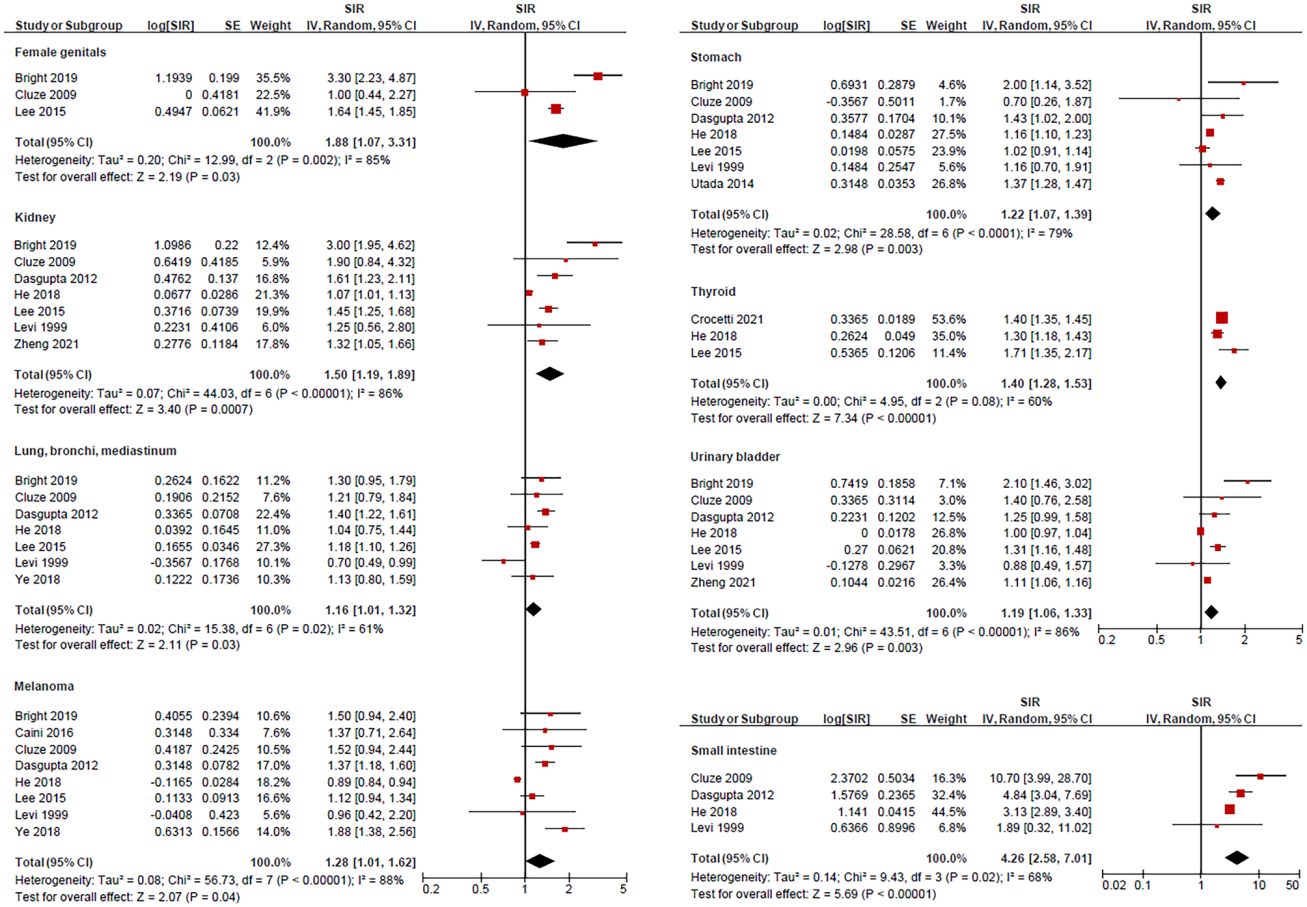
Second primary cancers with an increased risk following primary colorectal cancer including cancers from female genitals, kidney, thorax (lung, bronchi, and mediastinum), stomach, thyroid, urinary bladder, and small intestine as well as melanoma. The red squares and their sizes represent the effect sizes and weights of the included studies, respectively. The black diamonds and their sizes represent the pooled effect size and their 95% confidence intervals, respectively. The centre line of no effect runs through the value 1. Points to the right of the centre line (> 1) indicate an increased risk, whereas points to the left of the centre line (< 1) indicate a decreased risk

**Fig. 3:**
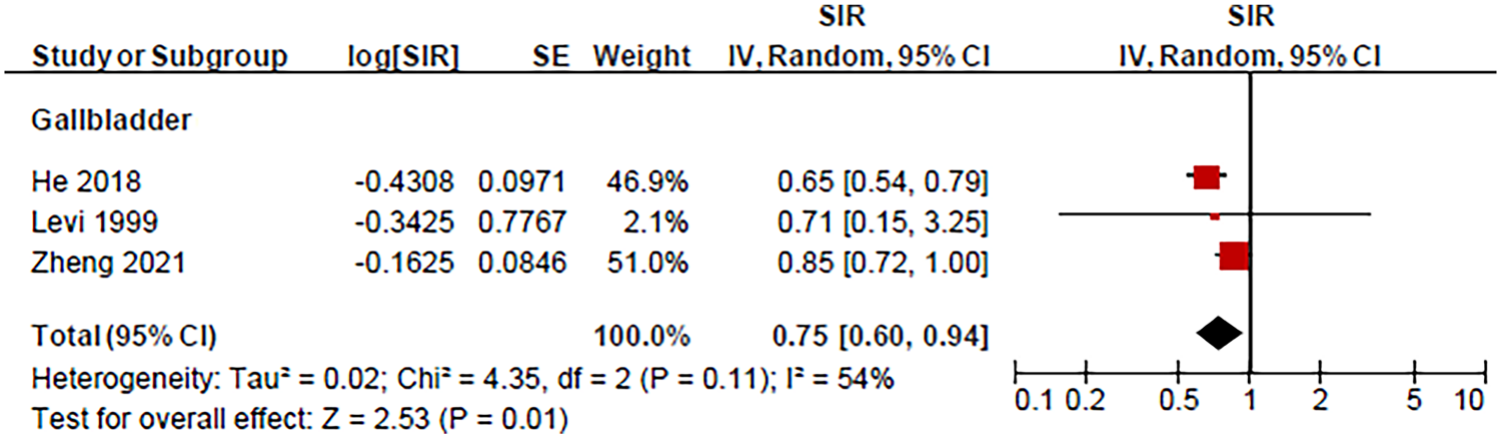
Second primary cancers with a decreased risk of following primary colorectal cancer: gall bladder cancer. The red squares and their sizes represent the effect sizes and weights of the included studies, respectively. The black diamond and its size represent the pooled effect size and its 95% confidence intervals, respectively. The centre line of no effect runs through the value 1. Points to the right of the centre line (> 1) indicate an increased risk, whereas points to the left of the centre line (< 1) indicate a decreased risk

We analysed the risk of extracolonic SPCs in patients with CRC among 13 studies reporting SIR (Table 1). The risk of several second primary cancers was significantly higher in patients with CRC compared with the general population’s risk of developing respective primary cancers. The risk of subsequent malignancies was greatest in the small intestine (pooled SIR = 4.26 (95% CI = 2.58–7.01; *p* < 0.0001)) from four studies [18, 21, 32, 33]; followed by the female genitals (1.88 (1.07–3.31; *p* = 0.03)) from three studies [16, 32, 34]; kidney (1.50 (1.19–1.89; *p* = 0.0007)) from seven studies [16, 18, 21, 32–34, 37]; thyroid (1.40 (1.28–1.53; *p* < 0.0001)) from three studies [33, 34, 36]; skin (melanoma) (1.28 (1.01–1.62; *p* = 0.04)) from eight studies [16, 18, 21, 30, 32–35]; stomach 1.22 ((1.07–1.39; *p* = 0.003)) from seven studies [16, 18, 21, 27, 32–34]; urinary bladder (1.19 (1.06–1.33; *p* < 0.0001)) from seven studies [16, 18, 21, 32–34, 37]; and lung, bronchi, and mediastinum (1.16 (1.01–1.32; *p* = 0.03)) from seven studies [16, 18, 21, 32–35]; Fig. 2. In contrast, there was a decreased risk of second primary gall bladder cancer (pooled SIR = 0.75; 95% CI = 0.60–0.94; *p* = 0.01) from three studies [21, 33, 38]; Fig. 3). There was no significant difference in the risk of second primary cancers of the prostate, pancreatic, ovaries, oesophagus, upper aerodigestive tract, liver and biliary tract, breast, cervix, uterus, and brain, nor in non-Hodgkin lymphoma, leukaemia, and myeloma (*p* > 0.05). The median follow-up years for each SPC are outlined in Table 2. According to the studies included in our analysis, the median follow-up time for SPCs was 4.2 years.

**Table 2:** Median follow-up periods for the second primary cancers included in the meta-analysis

**Second cancer**	**Median follow-up years (IQR)**
Urinary bladder	4.2 (3.8–12.1)
Brain	12.1
Breast	4.2 (3.8–12.1)
Cervix	5.7
Female genital	4.0 (3.5–16.8)
Gallbladder	7.3
Kidney	4.2 (3.8–12.1
Leukaemia	12.1
Liver, hepatic duct, and biliary	4.0 (3.5–7.3)
Lung, bronchus, and mediastinum	4.2 (3.8–12.1)
Melanoma	4.1 (3.9–9.7)
Myeloma	5.8
Non-Hodgkin lymphoma	10.5
Oesophagus	5.7 (3.6–14.4)
Ovary	7.3 (4.0–16.8)
Pancreas	4.1 (3.5–9.7)
Prostate	4.2 (3.8–12.1)
Small intestine	4.2 (3.5–7.3)
Stomach	4.2 (3.8–12.1
Thyroid	7.0 (4.0–7.3)
Upper aerodigestive tract	7.3 (3.5–16.8)
Uterus	4.2 (3.8–12.1)

### Publication bias

Heterogeneity was high in studies investigating the risk of urinary bladder, prostate, pancreatic, ovarian, stomach, kidney, lung, small intestine, upper aerodigestive tract, breast, uterine, thyroid, brain, female genital, and liver, hepatic duct, and biliary tract cancers, as well as melanoma, leukaemia, and myeloma. However, visual inspection of funnel plots showed no asymmetry which indicated no publication biases were present (Supplementary Fig. 1).

## Discussion

The findings of this systematic review and meta-analysis suggest that patients with CRC have a significantly higher risk of extracolonic SPCs than the general population, including cancers of the urinary bladder, female genitals, kidney, lung, bronchus and mediastinum, small intestine, stomach, and thyroid, as well as melanoma. The greatest risk was observed for SPC of the small intestine, more than fourfold, compared with the general population, while the increased risk was relatively less for other sites (less than twofold).

Previous studies have reported an increased risk of SPCs following CRC, particularly cancers of the urinary bladder [10, 12], kidney [10, 12], stomach [9, 10, 12], and the small intestine [9–12, 17], which are consistent with the results of our meta-analysis. Because of these findings, several possible mechanisms have been discussed. For example, some of the risks can be attributed to genetic predisposition, such as in cases of Lynch syndrome (hereditary non-polyposis colorectal cancer familial cancer syndrome), albeit rare [22, 23]. Another hypothesis pertains to the expectation that embryologically related tissues might respond in similar ways to environmental exposures or carcinogens and undergo comparable epigenetic changes conducive to tumourigenesis [17]. Indeed, the small intestine, stomach, urinary bladder, and lung share endoderm-derived epithelia and, therefore, may be linked in this manner. However, this was not supported by our observed decrease in the risk of second primary gall bladder cancer. Alternatively, specific mutations common to CRC and certain second primary malignancies may be responsible for the elevated risk. For instance, *v-raf murine sarcoma viral oncogene homologue* B1 (*BRAF*), one of the most frequently mutated protein kinase genes in human cancers, mutations are seen in melanoma, papillary thyroid carcinoma, and CRC [24]. In addition, in the follow-up of CRC, many of these SPCs of high prevalence (including cancers of the urinary bladder, female genitals, kidney, lung, bronchus and mediastinum, small intestine, and stomach) could be detected on the follow-up abdominal and chest computed tomography (CT) scan which may also contribute to higher pick-up rate of these SPCs.

Our study had several limitations. Misclassification of cancers in registry-based investigations may introduce over- or underestimation of SPC incidence rates. As we did not include metachronous CRC in our analysis, differentiating between SPCs and local recurrences was not an issue. Additionally, most studies reported attempts to prevent the inclusion of synchronous cancers by excluding subsequent cancers diagnosed within 2 months of the index CRC. There may have been some level of misclassification with respect to tumours arising in discrete sites, namely the lungs, bronchi, and mediastinum; upper aerodigestive tract; female genitals; and the liver, hepatic ducts, and biliary system. As such, we only pooled second cancers of discrete sites where explicitly consistent between individual studies for the robustness of our interpretations.

Although we anticipated and attempted to address heterogeneity in our planned analysis, it remained substantial for most pooled second cancers. This is likely due to epidemiological differences between studies, such as follow-up, the periods of time covered, changes in specific cancer demographics across time, varying selection criteria, and temporospatial differences in treatment modalities. Comparably moderate-to-high levels of heterogeneity have been previously observed and discussed in other meta-analyses on SPC [39, 40]. The heterogeneity in these meta-analyses can be largely attributed to differences in the magnitude of risk observed between studies. Ultimately, while we cannot be certain of the true magnitude of in the risk reported in the present study, our results are indicative of an increase in risk of specific second primary malignancies leading to further foci of research in the field.

There is lack of data to clearly document the effect of occurrence of SPC in the overall survival of patients with SPC. The survival of these patients is likely to be depending on the nature of the primary CRC and the SPC. If the CRC is of advanced stages and with residual cancer after resection as well as with mutation not amendable to target therapy, it is likely the survival is dismal and the impact of SPC on the survival is not apparent. On the other hand, if the CPC is of early stages and after curative resection, the survival of the patients with CRC is obviously affected and likely depend on the SPCs with high patients’ mortality and morbidity such as cancers of the thorax (lung, bronchus, and mediastinum) and melanoma [41, 42]. There are also SPCs such as from the urinary bladder, kidney, female genitals, small intestine, and stomach of similar diverse biological aggressiveness as CRC which will have impact of the survival on the patients. The only exception is in patients with SPC of thyroid cancer with is of increasing incidence worldwide. Thyroid cancer is mostly clinically indolent but could contribute to long-term morbidity of the patients with possibility of local recurrence, de-differentiation to clinical aggressive histological type, and thyroxine replacement therapy [43, 44].

In most clinical centres, the management of patients with CRC will be discussed in multidisciplinary team meeting and follow-up with standard protocols (such as radiology and endoscopic examinations) according to the prognostic parameters as well as personalised medical needs (such as comorbidity). Majority of the SPCs of relative higher prevalence could be detected by this means. Thus, awareness of the possibility of SPCs and adherence to protocols of follow-up of patients with CRC is important for best clinical practice. Nevertheless, we need more investigations to look at the length of intervals between CRC and SPCs. With the acknowledgement that the median follow-up time for SPCs from the studies included in our analysis was 4.2 years, it may be in some cases that SPCs may occur after the standard follow-up time for patients with CRC. Education on the patient and general practitioner of the issue should be of value to this group of patients. It is also important to have prospective clinical studies to address to the comorbidity issues and survival impacts in these patients.

## Conclusion

The findings of this systematic review and meta-analysis suggest that patients with CRC have an increased risk of extracolonic SPCs compared with the general population, including cancers of the urinary bladder, female genitals, kidney, lung, bronchus and mediastinum, small intestine, stomach, and thyroid, as well as melanoma. Future studies monitoring SPC risk in patients with CRC are warranted as there is a need to develop surveillance and management strategies to decrease the burden of subsequent malignancies within this expanding population.

## Supplementary information

Below is the link to the electronic supplementary material.

Supplementary file1 (DOCX 102 KB)

Supplementary file2 (DOCX 34 KB)
