# Supplementary material for: Risk of extracolonic second primary cancers following a primary colorectal cancer: a systematic review and meta-analysis
Source: Int J Colorectal Dis. 2022 Feb 12;37(3):541–51. doi: 10.1007/s00384-022-04105-x (PMC8885556; doi:10.1007/s00384-022-04105-x)
Supplement: Supplementary file 1 — Supplementary file1 (DOCX 102 KB) [file 384_2022_4105_MOESM1_ESM.docx]

**
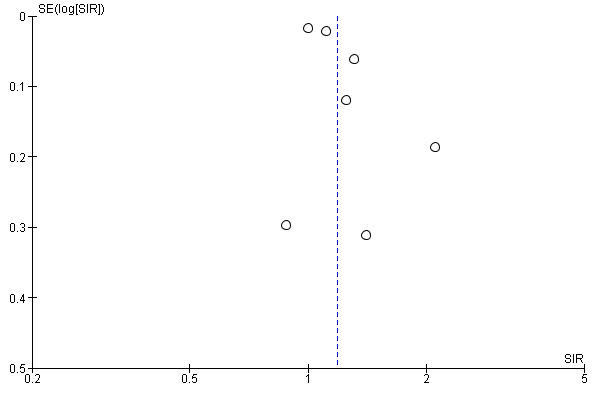
**

**Supplementary Figure 1.** Funnel plot for urinary bladder cancer.


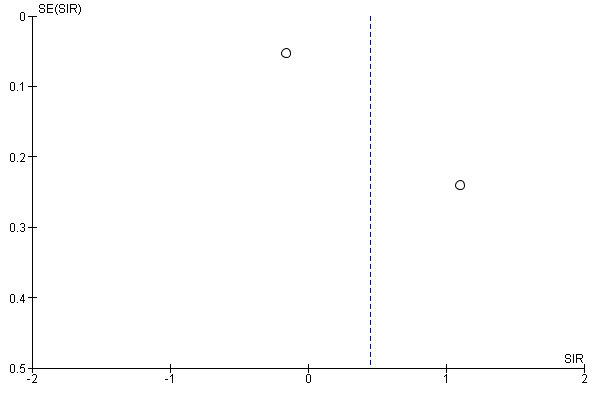


**Supplementary Figure 2.** Funnel plot for brain cancer.


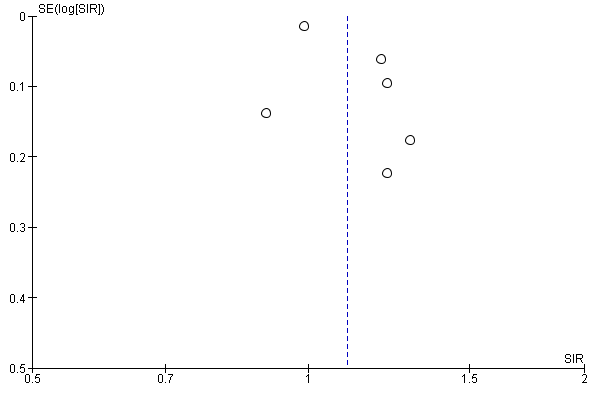


**Supplementary Figure 3.** Funnel plot for breast cancer.


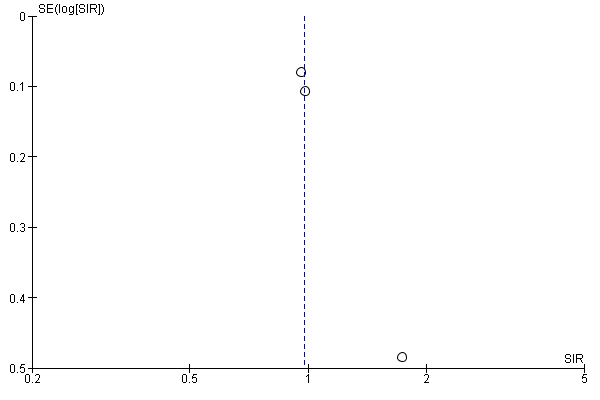


**Supplementary Figure 4.** Funnel plot for cervical cancer.


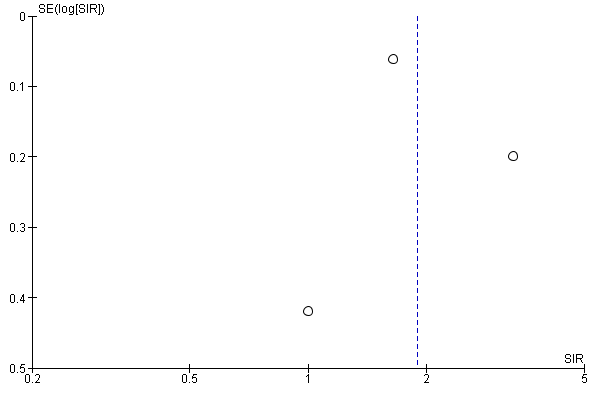


**Supplementary Figure 5.** Funnel plot for female genital cancer.


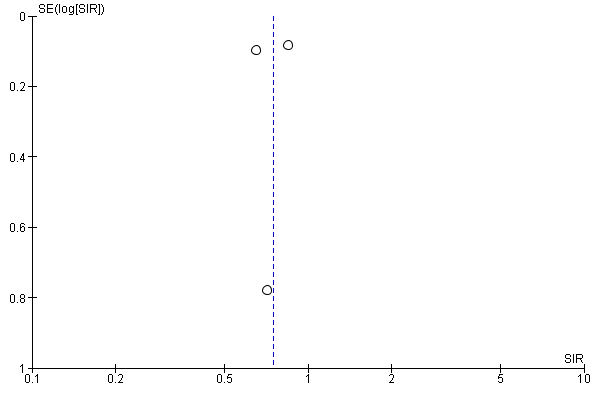


**Supplementary Figure 6.** Funnel plot for gallbladder cancer.


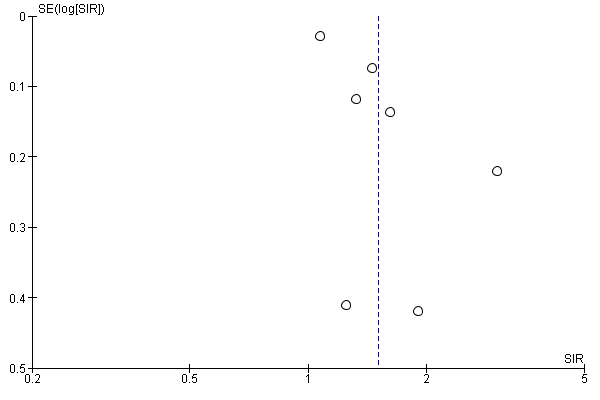


**Supplementary Figure 7.** Funnel plot for kidney cancer.


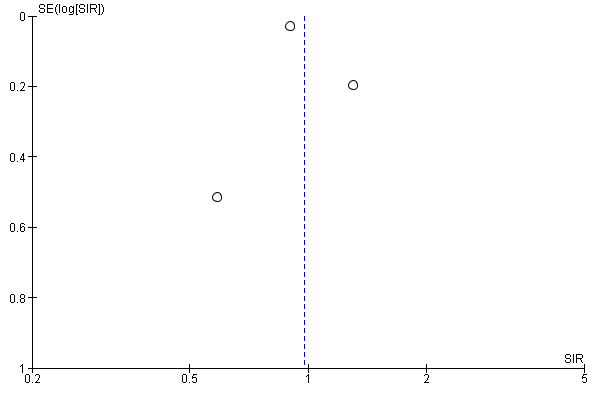


**Supplementary Figure 8.** Funnel plot for leukaemia.


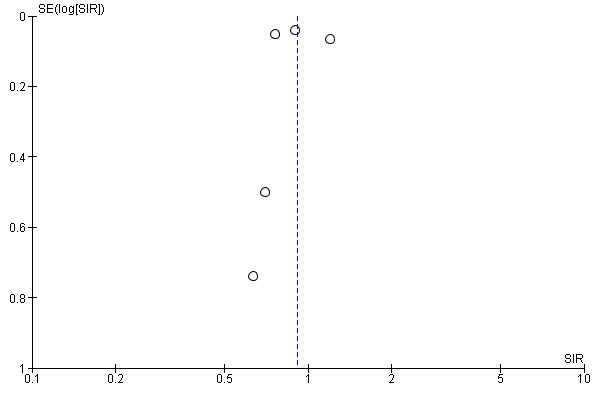


**Supplementary Figure 9.** Funnel plot for liver, hepatic duct, and biliary tract cancer.


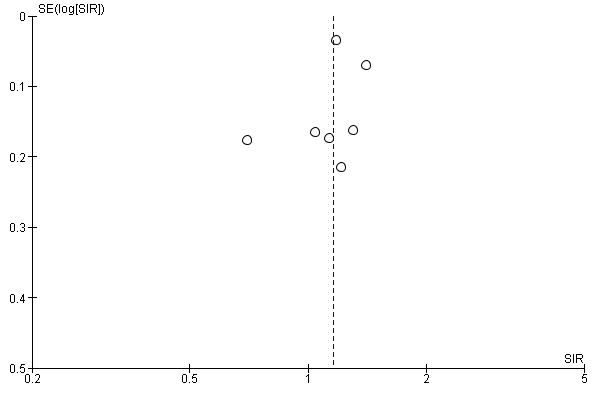


**Supplementary Figure 10.** Funnel plot for lung, bronchial, and mediastinal cancer.


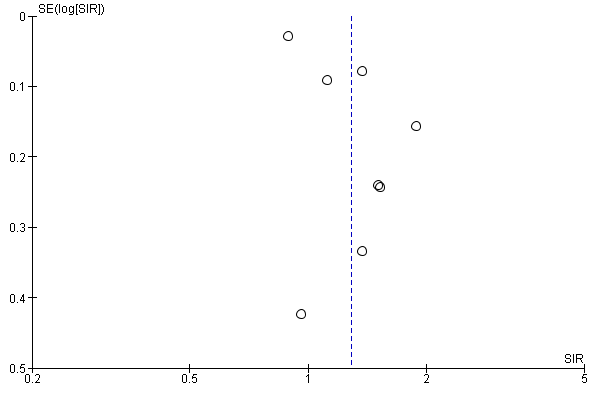


**Supplementary Figure 11.** Funnel plot for melanoma.


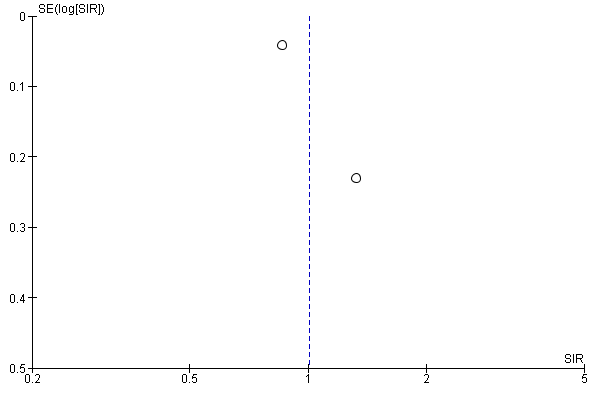


**Supplementary Figure 12.** Funnel plot for myeloma.


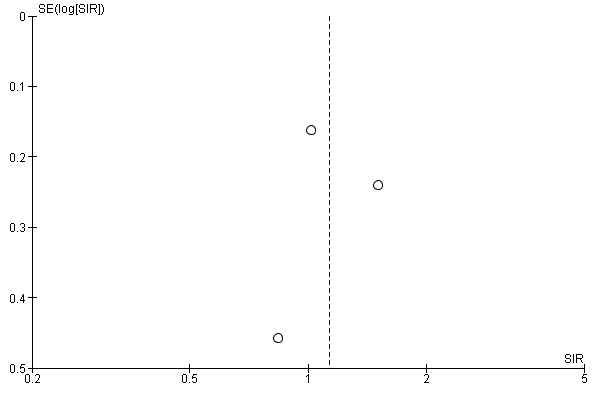


**Supplementary Figure 13.** Funnel plot for non-Hodgkin lymphoma.


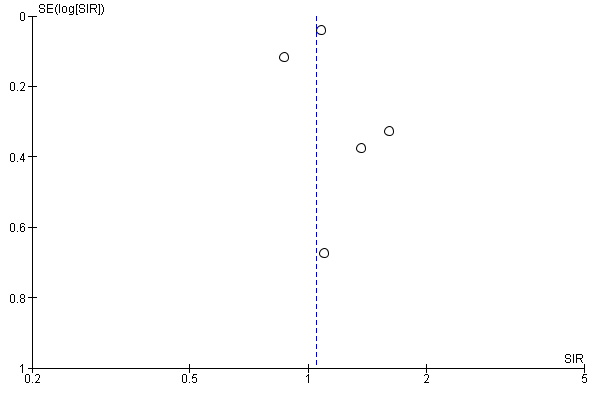


**Supplementary Figure 14.** Funnel plot for oesophageal cancer.


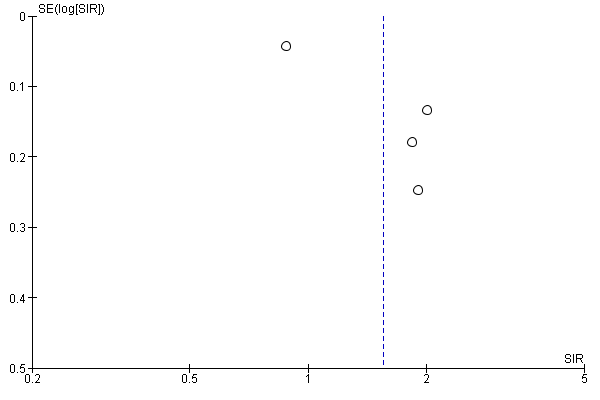


**Supplementary Figure 15.** Funnel plot for ovarian cancer.


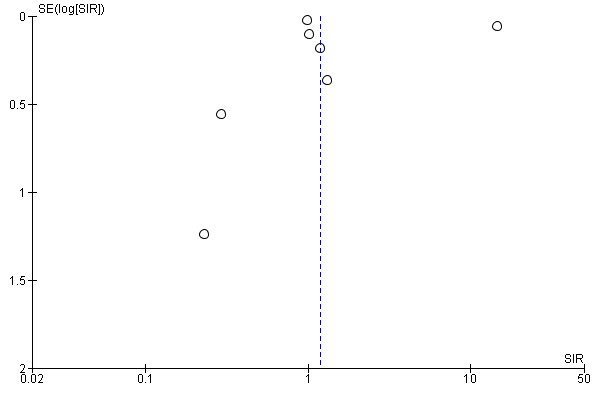


**Supplementary Figure 16.** Funnel plot for pancreatic cancer.


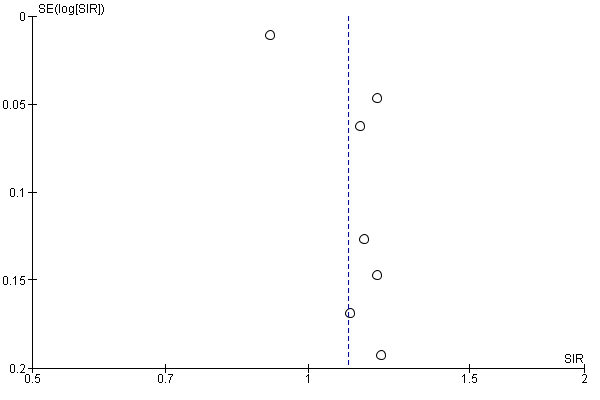


**Supplementary Figure 17.** Funnel plot for prostate cancer.


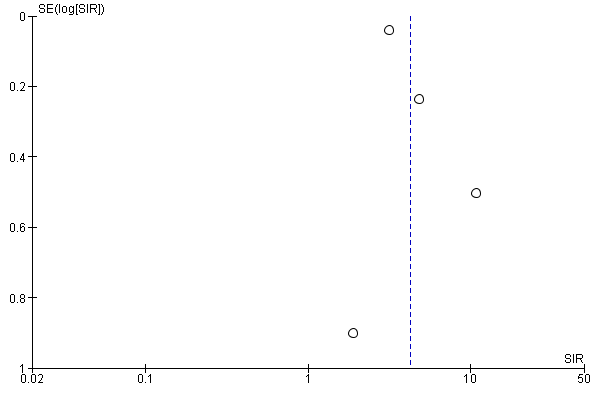


**Supplementary Figure 18.** Funnel plot for small intestinal cancer.


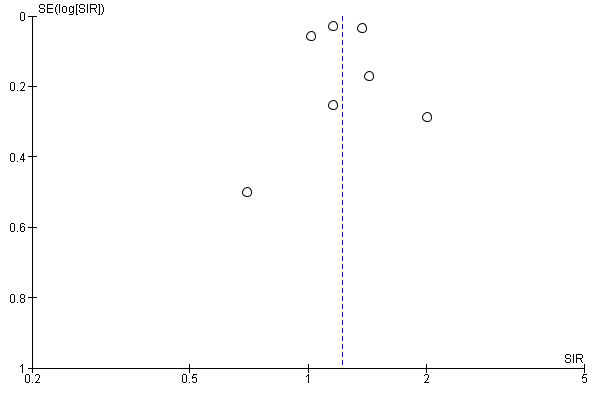


**Supplementary Figure 19.** Funnel plot for stomach cancer.


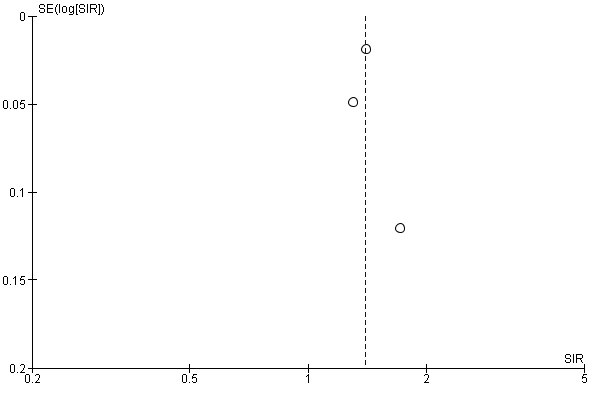


**Supplementary Figure 20.** Funnel plot for thyroid cancer.


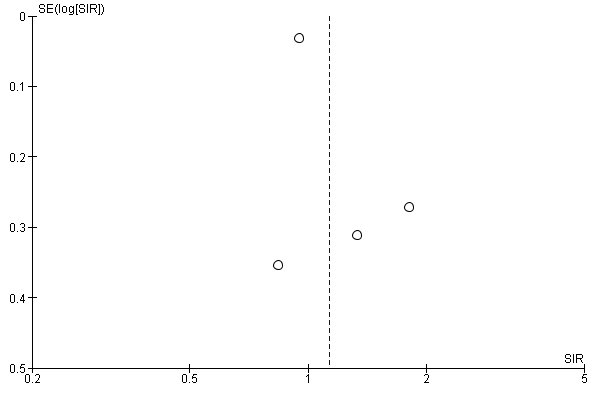


**Supplementary Figure 21.** Funnel plot for upper aerodigestive tract cancer.


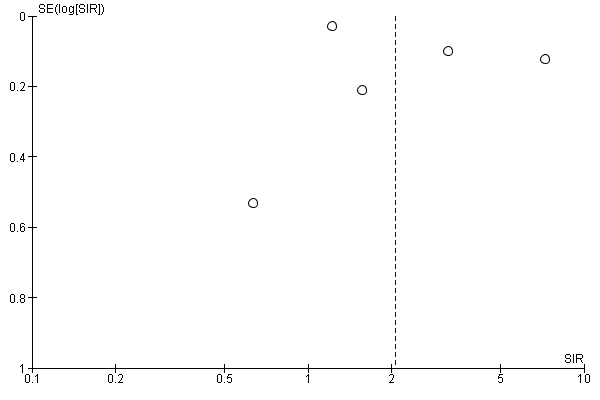


**Supplementary Figure 22.** Funnel plot for uterine cancer.
