# Supplementary material for: Risk of extracolonic second primary cancers following a primary colorectal cancer: a systematic review and meta-analysis
Source: Int J Colorectal Dis. 2022 Feb 12;37(3):541–51. doi: 10.1007/s00384-022-04105-x (PMC8885556; doi:10.1007/s00384-022-04105-x)
Supplement: Supplementary file 2 — Supplementary file2 (DOCX 34 KB) [file 384_2022_4105_MOESM2_ESM.docx]

**Supplementary Table 1.** Summary of calculations for expected cases in standardised incidence ratios (SIR) of included studies.

| **Study ID** | **Calculation for expected cases in SIR** | **Population/Registry, Country** |
| --- | --- | --- |
| Bright et al (2019)(16) | Multiplying the number of person-years accrued, stratified by gender, attained age (5-year bands), and calendar year (1-year bands) | General population, England and Wales |
| Caini et al (2016)(30) | The application of sex-, single year age, calendar year-, and country macro region- (North-western, North-eastern, Central, Southern and Islands) of specific cancer reference rates to the follow-up time accrued after melanoma diagnosis | European Institute of Oncology Database, Italy |
| Chung et al (2017)(31) | Cancer incidence rates to the corresponding general population | Korean Central Cancer Registry, South Korea |
| Cluze et al (2009)(32) | Site-, sex- and age (5-year intervals)-specific cancer incidence rates of the general population to the corresponding number of person–years at risk | Cancer Registry of Isère, France |
| Crocetti (2021)(36) | Multiplying the cumulative person‐years of observation by the specific incidence rates for the strata in which person‐years were distributed | Italian cancer registries, Italy. |
| Dasgupta et al (2012)(18) | Age- and sex-matched general population | Queensland Cancer Registry, Australia |
| He et al (2018)(33) | Age-specific, sex-specific, and race-specific incidence rates | Surveillance, Epidemiology, and End Results Program of the National Cancer in United States of America |
| Lee et al (2015)(34) | Age in 5-year intervals, sex, and calendar year by the specific stratified person-time variable | Taiwan National Health Insurance Database, Taiwan |
| Levi et al (1999)(21) | Site-, age- and calendar period-specific incidence rates multiplied by the corresponding number of person-years at risk | Vaud Cancer Registry, Switzerland |
| Utada et al (2014)(27) | Sex-, age group-, and year of diagnosis-specific incidence rates in the general population | Nagasaki Prefecture Cancer Registry, Japan |
| Ye et al (2018)(35) | Person-years at risk within strata multiplied by corresponding site-specific cancer incidence rates in the general population | Tasmanian Cancer Registry, Australia |
| Zheng et al (2021)(37) | Strata‐specific person years in patients with diagnosis of first primary bladder or upper urinary tract cancers, multiplied by strata‐specific incidence rates of the same SPC as FPC in the general population | Swedish Cancer Register, Sweden |
| Zheng et al (2021)(38) | The followed person-years after first primary cancer diagnosis, multiplied by the incidence of the same cancer as first primary cancer in the general population | Swedish Cancer Register, Sweden |
